# Supplementary material for: MetaRibo-Seq measures translation in microbiomes
Source: Nat Commun. 2020 Jun 29;11:3268. doi: 10.1038/s41467-020-17081-z (PMC7324362; doi:10.1038/s41467-020-17081-z)
Supplement: Supplementary file 10 — Supplementary Data 7 [file 41467_2020_17081_MOESM10_ESM.zip › File2/Confidence_High_Taxonomy/5855_out.krona.html]

Javascript must be enabled to view this page.

members
magnitude
magnitudeUnassigned
count
unassigned
taxon
rank

5855\_out

2

superkingdom
2
2

phylum
201174
2

1760
class
2

1
2037
order

1
2049
family

1069494
genus
1


SRS015590\_contig\_number\_16250
1661
species
1

85006
order
1

1
1268
family

genus
32207
1

species

SRS144090\_contig\_number\_1551
1739448
1
